# Spatiotemporal dissemination of ESBL-producing Enterobacterales in municipal sewer systems: a prospective, longitudinal study in the city of Basel, Switzerland

**DOI:** 10.3389/fmicb.2023.1174336

**Published:** 2023-05-12

**Authors:** Elena Gómez-Sanz, Claudia Bagutti, Jan A. Roth, Monica Alt Hug, Ana B. García-Martín, Laura Maurer Pekerman, Ruth Schindler, Reto Furger, Lucas Eichenberger, Ingrid Steffen, Adrian Egli, Philipp Hübner, Tanja Stadler, Lisandra Aguilar-Bultet, Sarah Tschudin-Sutter

**Affiliations:** ^1^Division of Infectious Diseases and Hospital Epidemiology, University Hospital Basel, University of Basel, Basel, Switzerland; ^2^Department of Clinical Research, University Hospital Basel, Basel, Switzerland; ^3^State Laboratory Basel-City, Basel, Switzerland; ^4^Rothen Laboratory, Basel, Switzerland; ^5^Applied Microbiology Research, Department of Biomedicine, University of Basel, Basel, Switzerland; ^6^Department of Biosystems Science and Engineering, ETH Zurich, Basel, Switzerland

**Keywords:** ESBL-producing Enterobacterales, wastewater, *Escherichia coli*, *Klebsiella pneumoniae*, spatiotemporal distribution

## Abstract

**Background:**

The contribution of community and hospital sources to the transmission of extended-spectrum β-lactamase producing Enterobacterales (ESBL-PE) remains elusive.

**Aim:**

To investigate the extent of community dissemination and the contribution of hospitals to the spread of ESBL-PE by exploring their spatiotemporal distribution in municipal wastewater of the central European city of Basel.

**Methods:**

Wastewater samples were collected monthly for two consecutive years throughout Basel, Switzerland, including 21 sites across 10 postcode areas of the city collecting either community wastewater (urban sites, *n* = 17) or community and hospital wastewater (mixed sites, *n* = 4). Presumptive ESBL-PE were recovered by selective culture methods. Standard methodologies were applied for species identification, ESBL-confirmation, and quantification.

**Results:**

Ninety-five percent (477/504) of samples were positive for ESBL-PE. Among these isolates, *Escherichia coli* (85%, 1,140/1,334) and *Klebsiella pneumoniae* (11%, 153/1,334) were most common. They were recovered throughout the sampling period from all postcodes, with *E. coli* consistently predominating. The proportion of *K. pneumoniae* isolates was higher in wastewater samples from mixed sites as compared to samples from urban sites, while the proportion of *E. coli* was higher in samples from urban sites (*p* = 0.003). Higher numbers of colony forming units (CFUs) were recovered from mixed as compared to urban sites (median 3.2 × 10^2^ vs. 1.6 × 10^2^ CFU/mL). *E. coli*-counts showed moderate correlation with population size (rho = 0.44), while this correlation was weak for other ESBL-PE (rho = 0.21).

**Conclusion:**

ESBL-PE are widely spread in municipal wastewater supporting that community sources are important reservoirs entertaining the spread of ESBL-PE. Hospital-influenced abundance of ESBL-PE appears to be species dependent.

## Introduction

Members of the order Enterobacterales are ubiquitous and form a major part of the microbiota of the human and animal intestine (Partridge, 2015). Some of them, such as *Escherichia coli* and the *Klebsiella, Enterobacter, Serratia, and Citrobacter* (KESC) group cause infections, including in the urinary tract, bloodstream, and respiratory tract, as well as intestinal and intra-abdominal infections (Pitout and Laupland, 2008; Qin et al., 2008). Extended-spectrum β-lactamase producing Enterobacterales (ESBL-PE) have been classified as serious and critical threats by public health authorities, such as the Centers for Disease Prevention and Control (CDC) and the World Health Organization (WHO) (Tacconelli et al., 2018; Centers for Disease Control and Prevention, 2019). Despite the implementation of infection prevention and control measures aiming to limit further spread in healthcare settings, the incidence especially of ESBL-producing *E. coli* and *Klebsiella pneumoniae* continues to increase worldwide (Jernigan et al., 2020).

Initially considered mainly hospital-acquired, the current epidemiology of ESBL-PE points to the increasing importance of community-based transmission (Pitout et al., 2005; Chong et al., 2018). Community-acquired ESBL sources include human-to-human transmission within or between households in the open community (Mughini-Gras et al., 2019), foodstuffs (Kluytmans et al., 2013), companion and farm animals (Mughini-Gras et al., 2019), and colonization resulting from global travel, especially to South Asia (Kuenzli et al., 2014). Water bodies worldwide have proven to be vast reservoirs of clinically significant antimicrobial resistant organisms (Aarestrup and Woolhouse, 2020). In line with this, ESBL-PE have been recovered in water samples from Swiss rivers and lakes (Zurfluh et al., 2013), possibly constituting an underappreciated route for further dissemination. Contamination of water bodies by anthropogenic discharges via biological spills, antibiotics and their active metabolites as well as urine and feces, is a significant contributor to the selection and widespread dissemination of antimicrobial resistance (AMR) (Hooban et al., 2021). With these premises, hospital and community sewage may represent relevant sources of ESBL-PE. Yet, the potential variable contribution of different clinically relevant species among Enterobacterales, such as *E. coli* and *K. pneumoniae*, remains to be elucidated.

Although humans are the main source of community-acquired carriage of ESBL-producing species such as *E coli*, transmission within the community alone might not be self-sustaining without mobilization to and from non-human sources (Mughini-Gras et al., 2019). Hence, detailed knowledge on the specific contribution of different sources of ESBL-PE is essential for an efficient allocation of resources and as a basis for tailored infection prevention and control strategies. Most recently, environmental surveillance of municipal wastewater has been shown to be most useful in tracking emerging bacteria and viruses and monitoring their changing epidemiology (Hendriksen et al., 2019; Bagutti et al., 2022).

To obtain a deeper understanding of ESBL-PE circulation in both hospital and community settings, we explored the spatiotemporal distribution of ESBL-PE in urban wastewater collected throughout the sewage system of the city of Basel, Switzerland, representing both community and hospital sources. We further determined the abundance of presumptive ESBL-producing *E. coli* and KESC group to explore bacterial load distribution across the municipal sewage system as well as correlations with socio-economic population determinants and meteorological data.

## Materials and methods

### Study design

This prospective and cross-sectional longitudinal study (Stadler et al., 2018) (pre-registered on ClinicalTrials.gov; identifier NCT03465683) was performed in the city of Basel, Switzerland, over a 24-month period. Municipal wastewater representing the city’s sewage system and covering 44% of the population of Basel was sampled and analyzed for the presence of ESBL-PE. Sampling sites were categorized based on the wastewater sources received, as urban (representing the community without wastewater from healthcare settings) and mixed (representing both community and healthcare settings).

### Setting

Basel, a central European city organized in 10 different postcode areas, has a population of around 180,000 inhabitants. Its healthcare system comprises 14 hospitals. Among these, the University Hospital Basel, a > 600-bed tertiary care academic center that admits over 35,000 patients per year, constitutes the largest hospital in the city. The sewage system of Basel is an underground sewer network that receives sanitary hospital wastewaters without pre-treatment in accordance with the current national legislation.

### Sampling and data sources

**Figure 1:**
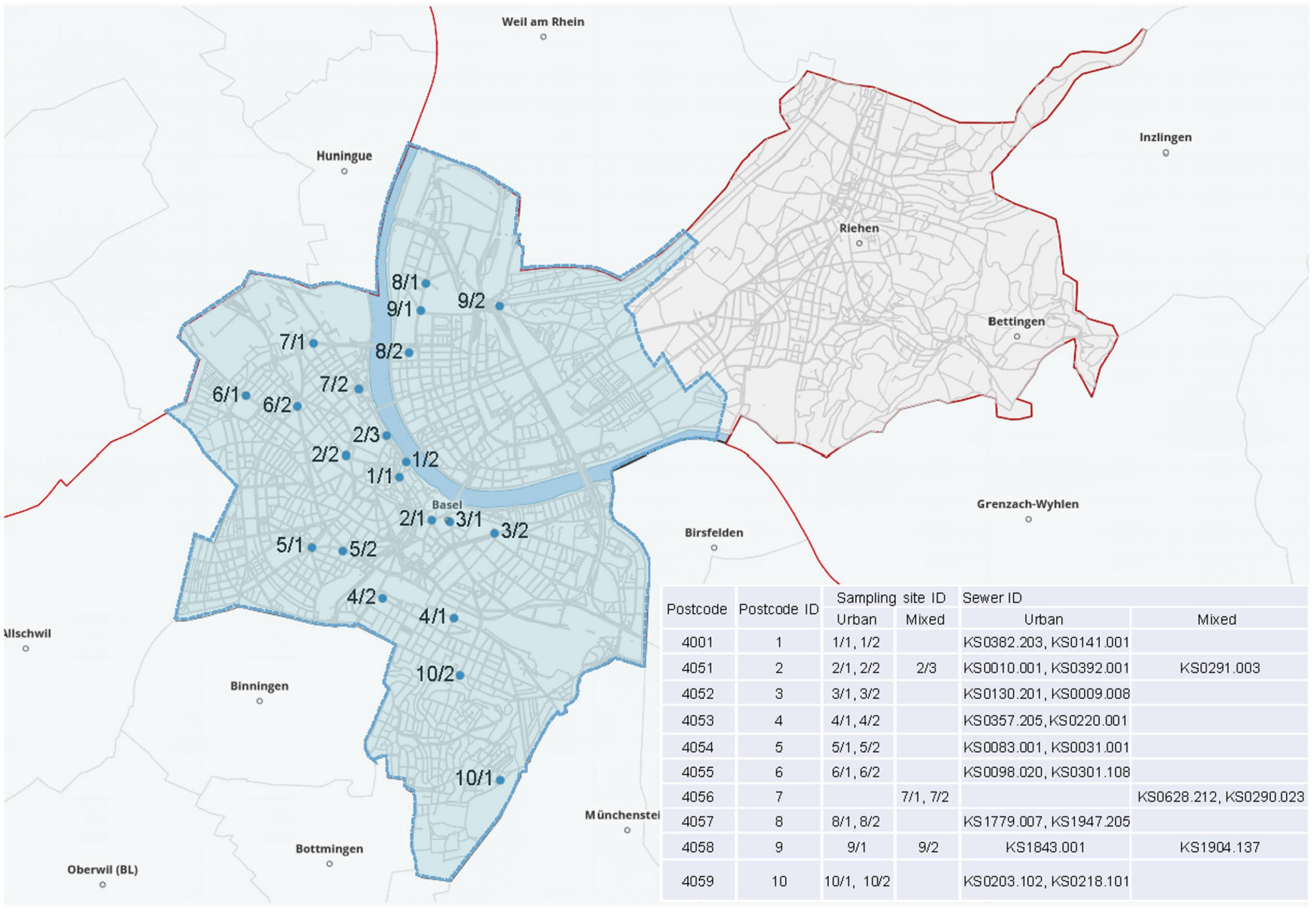
Distribution of the wastewater sampling sites across the city of Basel. The coding 1–10/1–3 refers to postcode area (1–10) and sampling site (1–3) specifying the district. The blue background delimits the city of Basel. Copyright by Google Maps.

Wastewater samples were collected monthly for two consecutive years (June 2017 until June 2019), except for July 2018 during which sampling could not be performed. Therefore, an additional month was included at the end of the study (June 2019). Twenty-one sampling sites (two per postcode area) best reflecting the wastewater of the entire city were chosen. Three sampling sites (4,056/1, 4,056/2, and 4058/2) compile both community and hospital wastewater. One sampling site (4,051/3) was included to capture wastewater from the University Hospital Basel. This sampling point collects community water and 35–40% of the hospital sewage. The remaining 17 sites receive wastewater from the general population. The specific location of the sampled sewers is displayed in Figure 1. Supplementary Table S1 denotes the sites receiving hospital water, displays population size per postcode area, and defines the catchment area per sampling point. The samples were collected directly from the sewage system by the Civil Engineering Department of the Canton of Basel-Stadt following the specific recommendations of the World Health Organization (WHO).^1^

The population size covered per sampling site was calculated. For this, the Civil Engineering Department drew the sewer system per sampling point on the Geoviewer 5.4 platform and gathered the street addresses covered for each of the sewer catchment areas (Supplementary Table S1). The Statistical Office of the canton of Basel-Stadt provided the number of inhabitants for each address/catchment area. The average population per sampling site was considered as a mean of the sampling years (2017–2019). The overall population covered was calculated based on the average population of Basel along the sampling years (Supplementary Table S1).

Information on socio-economic, population-based data and other key features of the individual districts was provided by the Statistical Office of the canton of Basel Stadt and is publicly available.^2^ We obtained meteorological data for Basel overall from the validated weather simulation archive of meteoblue.^3^

### Definitions

District refers to the city area covered by two sampling sites that mostly represent a given postcode area. Generated districts are not equivalent to a postcode area because the sewer system did not allow for choosing sampling sites comprising the exact area of the postcode. ESBL-PE refer to phenotypically confirmed ESBL-producing Enterobacterales. Presumptive ESBL-producing isolates refer to those grown in selective media without or prior to an ESBL confirmation test. Meteorological seasons were classified as follows: Winter (December–February), Spring (March–May), Summer (June–August), Autumn (September–November). Calendar months span from January (1) to December (12). Sampling rounds span from 1 (June 2017) to 24 (June 2019, as July 2018 is missing).

### Wastewater sample collection, processing, and isolation of presumptive ESBL-PE

Samples were collected in sterile 25-mL Falcon tubes (VWR, Dietikon, Switzerland) and transferred to the laboratory within 6 h for processing. Subsequently, 1 mL of wastewater sample was diluted into 9 mL of Enterobacteriaceae Enrichment (EE) Broth (Oxoid, Thermo Fisher Diagnostics, Pratteln, Switzerland) and incubated at 37° C overnight. This enrichment step was pursued to increase sensitivity, hence, to recover as many and phenotypically diverse ESBL-producing Enterobacterales as possible. The enrichments were then diluted 1/100 and 1/1,000 in 1 X Phosphate-Buffered Saline (PBS) medium and 100 μL of both dilutions were spread onto Brilliance™ ESBL plates (Oxoid) and incubated at 37°C for 24 h. This chromogenic medium provides presumptive identification of ESBL-producing *E. coli* (blue/pink) and the KESC group (green). If no colonies grew, 100 μL of the enrichment was streaked onto the plates in undiluted form and incubated at 37° C for 24 h and the initially spread plates were checked after 48 h incubation. One to three colonies per color and/or morphology were chosen and further isolated onto new Brilliance™ ESBL plates.

### Species identification, ESBL confirmation, and selection criteria

Species identification of all isolates was performed in duplicate on fresh cultures by Matrix-Assisted Laser Desorption/Ionization Time-Of-Flight mass spectrometry (MALDI-TOF MS). The isolates were fixed on the MALDI-TOF MS plate using a CHAC matrix (alpha-cyano-4-hydroxycinnamic acid) (Ziegler et al., 2015) and processed using the MALDI-TOF MS Axima^™^ Confidence (Shimadzu-Biotech, Reinach, Switzerland) and the SARAMIS^™^ Database (Spectral Archive and Microbial Identification System, AnagnosTec, Potsdam-Golm, Germany) (Welker and Moore, 2011). Both readouts needed to be consistent for bacterial-species taxonomy assignment. Therefore, no clear identification applied when either both measurements were ambiguous (i.e., below 75% identification) or one of the two duplicates gave no result. If no clear identification was achieved, the spectra were matched against the ribosomal marker-based database PAPMID™ (Mabritec AG, Riehen, Switzerland) (Kassim et al., 2017) applying the same performance criteria. Isolates identified as Enterobacterales and forming colonies of different color and/or morphology were stored in glycerol/Triptic Soy Broth (TSB) (1:1) at −80°C for downstream analysis. Isolates not identified as Enterobacterales, those with unclear or inconsistent identification, and duplicates with the same identification as other selected isolates per sample were discarded and considered as not fulfilling the study inclusion criteria. It should be noted that our MALDI-TOF database at the time of identification could not discriminate some species of the *K. pneumoniae* complex (including *K. quasipneumoniae*, *K. variicola*, and *K. quasivariicola*). Likewise, *E. cloacae* isolates may include other species of the complex, such as *E. ludwigii* and *E. kobei*, as they were not included in the database (Supplementary Table S2; Godmer et al., 2021; Voellmy et al., 2022).

ESBL confirmatory testing was performed and evaluated on all stored isolates according to the Clinical & Laboratory Standards Institute (CLSI) guidelines (Clinical and Laboratory Standards Institute CLSI, 2009) and using the Total ESBL Confirm Kit (Rosco Diagnostica, Axon Lab, Baden, Switzerland). The type of cephalosporin tested (cefotaxime and ceftazidime, cefepime) depended on the species and was applied according to the manufacturer’s instructions. For *Raoultella* spp., enclosed within the Klebsiella genus till 2001 (Appel et al., 2021), cefotaxime and ceftazidime were used. The plates were incubated at 35°C for 18 ± 2 h. ESBL production was confirmed when the zone of inhibition of the disk with cephalosporine plus clavulanate was ≥5 mm larger than the one around the cephalosporine alone. All isolates fulfilling these criteria were designated ESBL-PE.

### Quantification of presumptive ESBL-producing isolates and water temperature measurement

From round 14 (13/08/2018) on, presumptive ESBL-producing isolates were quantified. For this, we included three additional consecutive samplings after the end of the study period (16/07/2019; 19/08/2019; 24/09/2019), resulting in 14 months of quantification. Samples were kept in an insulated box to avoid warming. Samples were plated directly without previous enrichment step. For this, 100 μL of the samples were spread directly and in a 1:10 dilution (TSB) on Brilliance™ ESBL agar and colonies were counted after 24 h incubation at 37°C. Enumeration was performed by counting all colonies according to their color as described before. For the quantification analysis, we distinguished two chromogenic groups according to the manufacturer’s manual: *E. coli* as blue/pink and the KESC group as green colonies. We used these categories to assign *E. coli* or KESC group species to the appearing colonies. No further identification was pursued. From round 16 (16/10/2018) on (including the three additional samplings) the wastewater temperature at sampling was recorded for a total of 12 months.

### Statistical analyses

Microbiological characteristics were assessed both on an isolate-level and a collapsed wastewater sample-level. Respective differences in distributions were calculated using Chi-squared, Fisher’s exact, and Wilcoxon rank sum/Kruskal-Wallis tests—as appropriate. Missing data is indicated throughout. Correlation analyses of sample, socio-economic and meteorological parameters were performed using the Spearman’s rank correlation coefficients (rho) ranging from −1 to 1. Meteorological data were standardized across time and merged on a day-level for Basel overall. Socio-economic/population characteristics data were collapsed on a district-year level (medians) by weighting, where appropriate, for population size. Correlation strengths (rho) were defined as no correlation (0), very weak (0.01–0.19), weak (0.20–0.39), moderate (0.40–0.59), strong (0.60–0.79), and very strong (0.80–1.00). To visualize the strength of the relationships between the different variables, the matrix information were plotted using heatmaps (Stata packages “heatplot” (Jann, 2019), “palettes” and “colrspace”). Intracluster correlation was marginal overall and was therefore not considered in the explorative hypothesis tests. All analyses were performed on a multicore system with Stata/MP version 16 (Stata Corp., College Station, Texas, United States). All reported *p*-values are two-sided.

## Results

### ESBL-PE positive sample confirmation and ESBL-PE species identification

**Figure 2:**
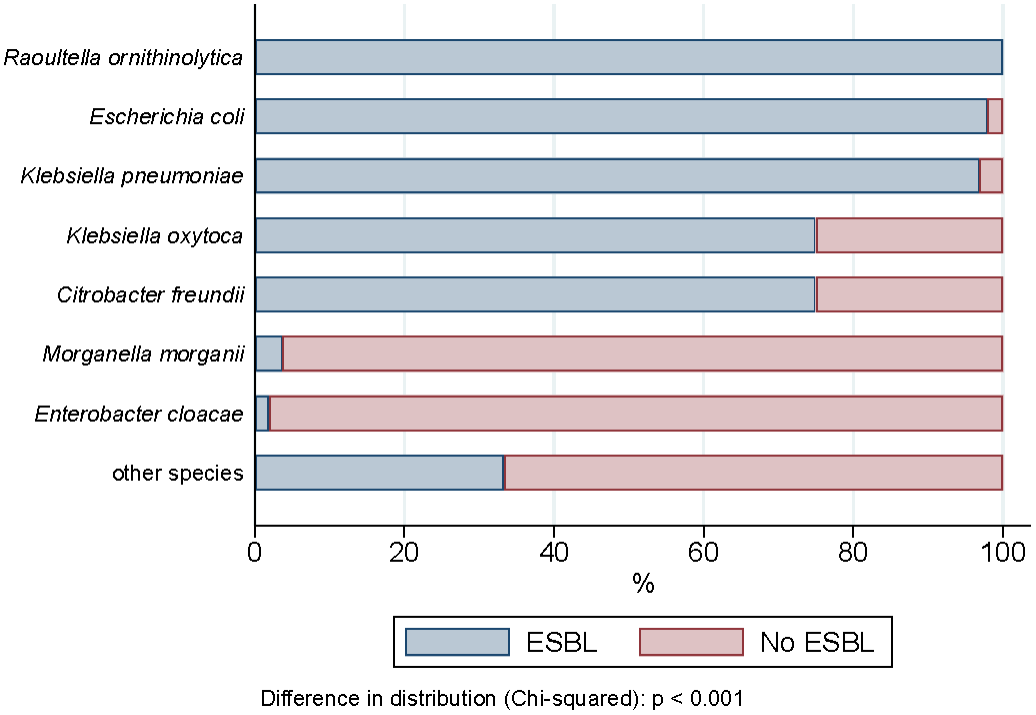
Phenotypic ESBL distribution across the enterobacterales recovered (*n* = 1,461).

ESBL-PE were recovered from 94.6% (477/504) of all wastewater samples collected during the 2-year sampling period. In total, 1993 isolates were recovered, of which 1,461 met the study inclusion criteria (see “Species identification, ESBL confirmation and selection criteria” section). Supplementary Table S3 details all isolates recovered across samples (space and time), wastewater source, species identified and ESBL profile. Among them, ESBL-production was phenotypically confirmed for 91.3% (1,334/1,461). *Escherichia coli* was by far the most common species identified, accounting for 85.5% of all ESBL-PE (1,140/1,334), followed by *Klebsiella pneumoniae* (11.5%, 153/1,334) (Table 1). Twenty-eight (2%) isolates belonged to other species of the KESC group (10 *Klebsiella* spp., 16 *Citrobacter* spp., and 2 *Enterobacter* spp.). Thirteen (<1%) additional ESBL-producing isolates belonged to *Raoultella ornithinolytica* (11/1,334), *Morganella morganii* (1/1,334) and *Proteus mirabilis* (1/1,334). No additional *Escherichia* species, other than *E. coli* were identified. The proportion of phenotypic ESBL-confirmation positive tests differed across presumptive ESBL-PE (i.e., isolates growing on Brilliance™ ESBL plates), ranging from 97.9% for *E. coli* (1,140/1,164), 96.8% for *K. pneumoniae* (153/158) and 100% for *R. ornithinolytica* isolates (11/11) to less than 1% for *Enterobacter cloacae* (1/52) and *Morganella morganii* (1/27) (Figure 2, for detailed values see Supplementary Table S4).

**Table 1:** ESBL-producing species identified across the ESBL-PE isolates (*n* = 1,334), isolates recovered per species and relative abundance.

ESBL species recovered	No. of ESBL positive	%
*Citrobacter amalonaticus*	2	0.15
*Citrobacter freundii*	12	0.90
*Citrobacter koseri*	1	0.07
*Citrobacter* sp.	1	0.07
*Enterobacter cloacae*	1	0.07
*Enterobacter xiangfangensis*	1	0.07
*Escherichia coli*	1,140	85.46
*Klebsiella aerogenes*	1	0.07
*Klebsiella oxytoca*	9	0.67
*Klebsiella pneumoniae*	153	11.47
*Morganella morganii*	1	0.07
*Proteus mirabilis*	1	0.07
*Raoultella ornithinolytica*	11	0.82

### Spatial distribution

**Figure 3:**
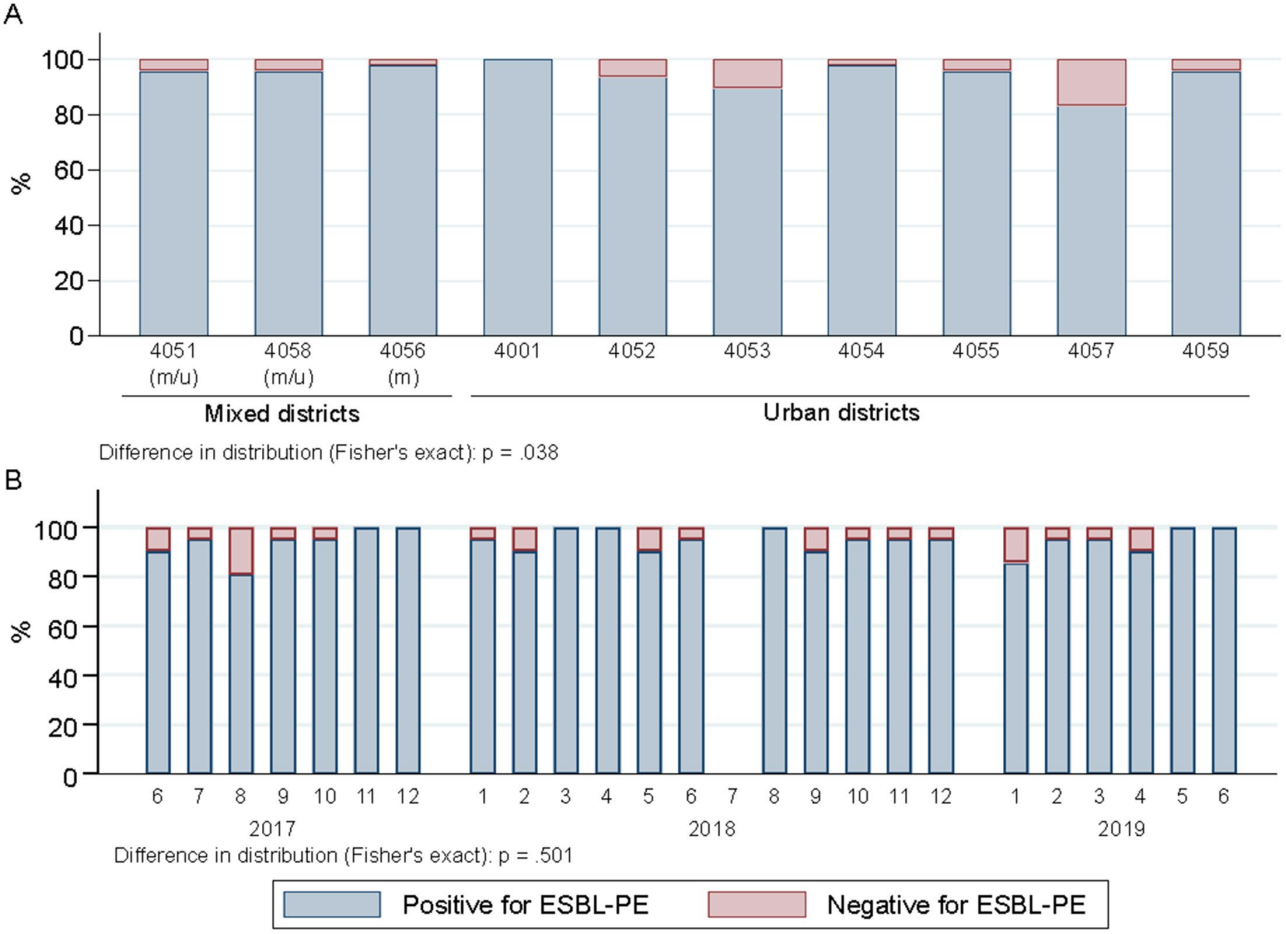
Spatiotemporal distribution of ESBL-PE across samples (*n* = 504). **(A)** Spatial distribution of ESBL-positive samples across city districts (part of a postcode area). Districts are ordered according to the mixed and urban site distribution (two sites per district, except for 4,051 three). m/u, mixed district comprising one site collecting hospital and urban (m) wastewater and other contributing only with urban wastewater (u); m, district collecting mixed water in both sampled sites. Numerator, number of ESBL samples in given district; denominator, 48 samples per district each except 72 for 4,051. **(B)** Temporal distribution of ESBL-positive samples across sampling months. 1–12 refers to the calendar months January (1) to December (12). Numerator, number of ESBL samples in given sampling point; denominator, 21 samples each. Samples negative for ESBL-PE enclose the following strata: samples with no growth (*n* = 14), samples with growth but no Enterobacterales (*n* = 10) and samples with Enterobacterales but ESBL negative (*n* = 3).

**Figure 4:**
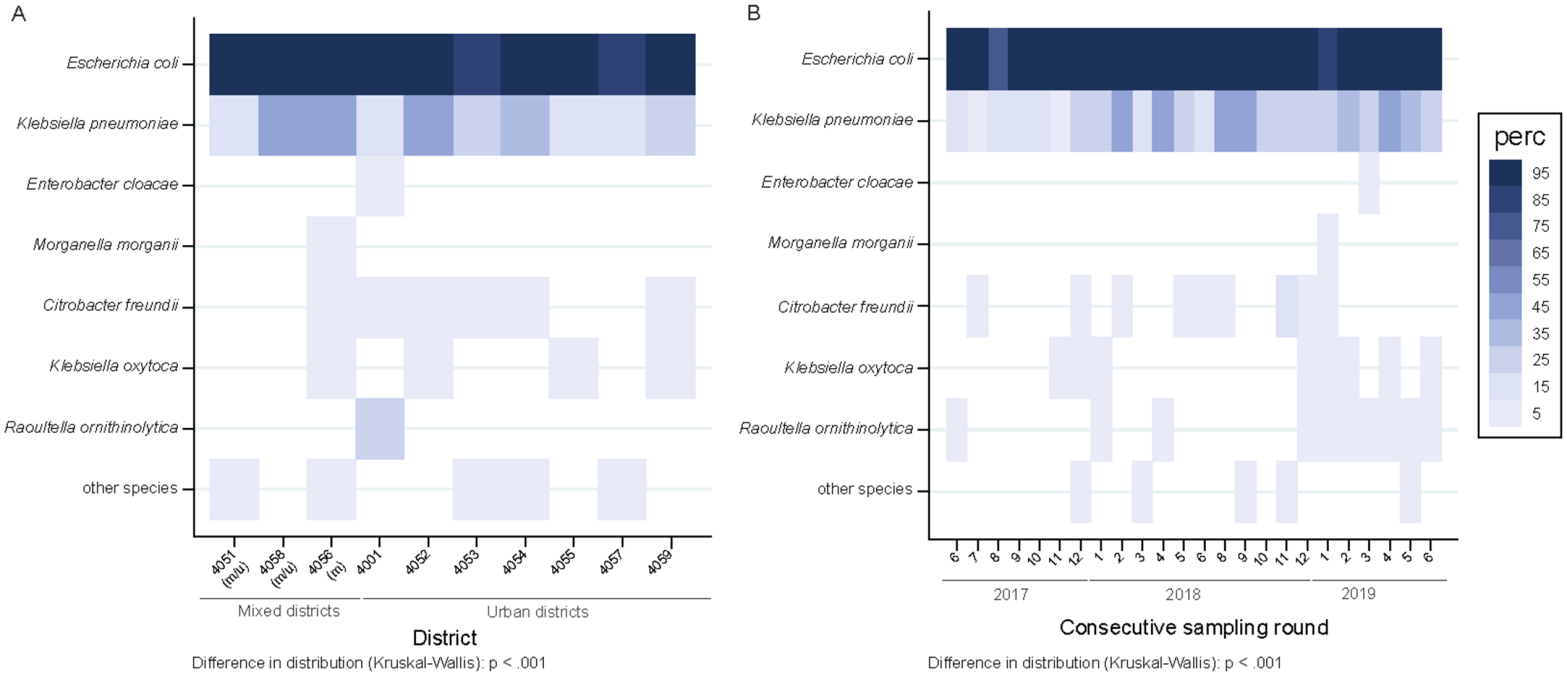
Spatiotemporal distribution of ESBL-producing species across samples (*n* = 504 overall), represented as percentage of samples positive for ESBL-PE. **(A)** Spatial, sample-level ESBL species distribution across city districts. Districts are ordered according to the mixed and urban site distribution (two sites per district, except for 4,051 three). m/u, mixed district comprising one site collecting hospital and urban (m) wastewater and other contributing only with urban wastewater (u); m, district collecting mixed water in both sampled sites. Numerator, number of samples with ≥1 ESBL isolate/s of a given species with at least 10 isolates (representing 0.75% of all isolates tested; *n* = 1,461) in a given district; denominator, 48 samples per district each except 72 for 4,051. Postcodes are ordered according to mixed and urban site distribution. Species are ordered according to the number of recovered isolates per species. **(B)** Temporal, sample-level distribution across consecutive sampling rounds. Numbers 1–12 refer to the calendar months January (1) to December (12). Numerator, number of samples with ≥1 ESBL isolates of a given species in a specific sampling point; denominator, 21 samples each.

The percentage of wastewater samples harboring ESBL-PE differed across districts (*p* = 0.038), mainly due to two areas (4,057 and 4,053), both with lower proportions of ESBL-PE positive samples (Figure 3A). The proportions of wastewater samples positive for individual species of ESBL-PE differed across districts (*p* < 0.001) (Figure 4A). Of note, we only display species with ≥1 ESBL isolate/s representing ≥0.75% of all isolates tested (≥ 10 isolates of 1,461) in a given postcode area. ESBL-producing *E. coli* and *K. pneumoniae* were recovered from all sampling sites, with *E. coli* consistently predominating across all districts (Figure 4A and Supplementary Table S3). ESBL-producing *R. ornithinolytica*, *E. cloacae*, and *M. morganii* were exclusively recovered from wastewater of unique districts (Figure 4A).

### Temporal distribution

The proportion of samples positive for ESBL species did not differ between sampling rounds (1–24) or calendar months (1–12) (*p* = 0.501) (Figure 3B). However, temporal differences in the detection of different ESBL-producing species were observed (*p* < 0.001) (Figure 4B) (criteria for species represented as indicated above). ESBL-producing *E. coli* and *K. pneumoniae* were the only species recovered throughout the entire sampling period (Figure 4B) with ESBL-producing *E. coli* consistently predominating in all sampling rounds (present in >90% of the samples; 475/504), while ESBL-producing *K. pneumoniae* was detected in close to one third of the samples (143/504) (Figure 4B). ESBL-producing *R. ornithinolytica* was consistently recovered along ten sampling rounds in the same district. Instead, ESBL-producing *E. cloacae* and *M. morganii* isolates were only recovered once (Figure 4B).

### Comparative analysis of mixed and urban sites

**Figure 5:**
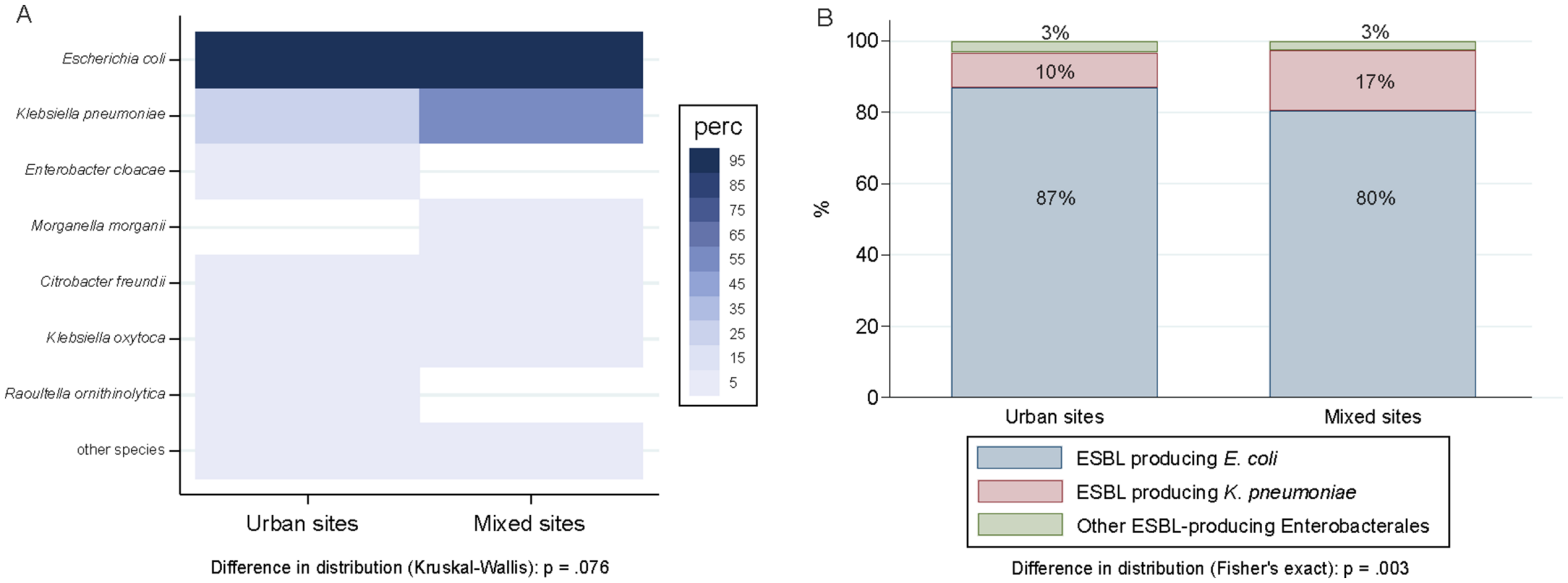
Distribution of ESBL-producing species across compiled urban (*n* = 96) and mixed sites (*n* = 408). **(A)** Sample-level distribution of ESBL-PE as percentage of positive samples. Species are ordered according to the number of isolates per species. Numerator, number of samples with ≥1 ESBL isolate of that given species; denominator, 408 urban samples, 96 mixed samples. Of note, only species with ≥1 ESBL isolate/s representing ≥0.75% of all isolates tested (≥ 10 isolates of 1,461) are displayed. **(B)** Distribution of ESBL-producing isolates (*n* = 1,334) clustered per species as *E. coli* (1140), *K. pneumoniae* (153), and others (41). Numerator, number of ESBL-producing isolates of that given species; denominator, 1,021 isolates in urban site, 313 in mixed sites.

The proportion of wastewater samples harboring ESBL-PE did not differ between mixed (97.9%; 94/96) and urban (93.9%; 383/408) sampling sites (*p* = 0.135) (Supplementary Table S5). The distribution of different ESBL-producing species between mixed and urban sites was similar (*p* = 0.076), except for *K. pneumoniae*, with an increased proportion of detection in mixed sites (50.0% vs. 22.3% in urban sites) (Figure 5A). Among all ESBL isolates recovered during the study period, the proportion of *K. pneumoniae* isolates was higher in wastewater samples from mixed sites (16.9%; 53/313) as compared to samples recovered from urban sites (9.8%; 100/1021) (*p* < 0.001). Contrary, the proportion of ESBL-producing *E. coli* was higher in samples from urban sites (87.0% vs. 80.1%) (Figure 5B) (*p* = 0.006).

### Quantification of presumptive ESBL chromogenic groups and correlation analyses

**Figure 6:**
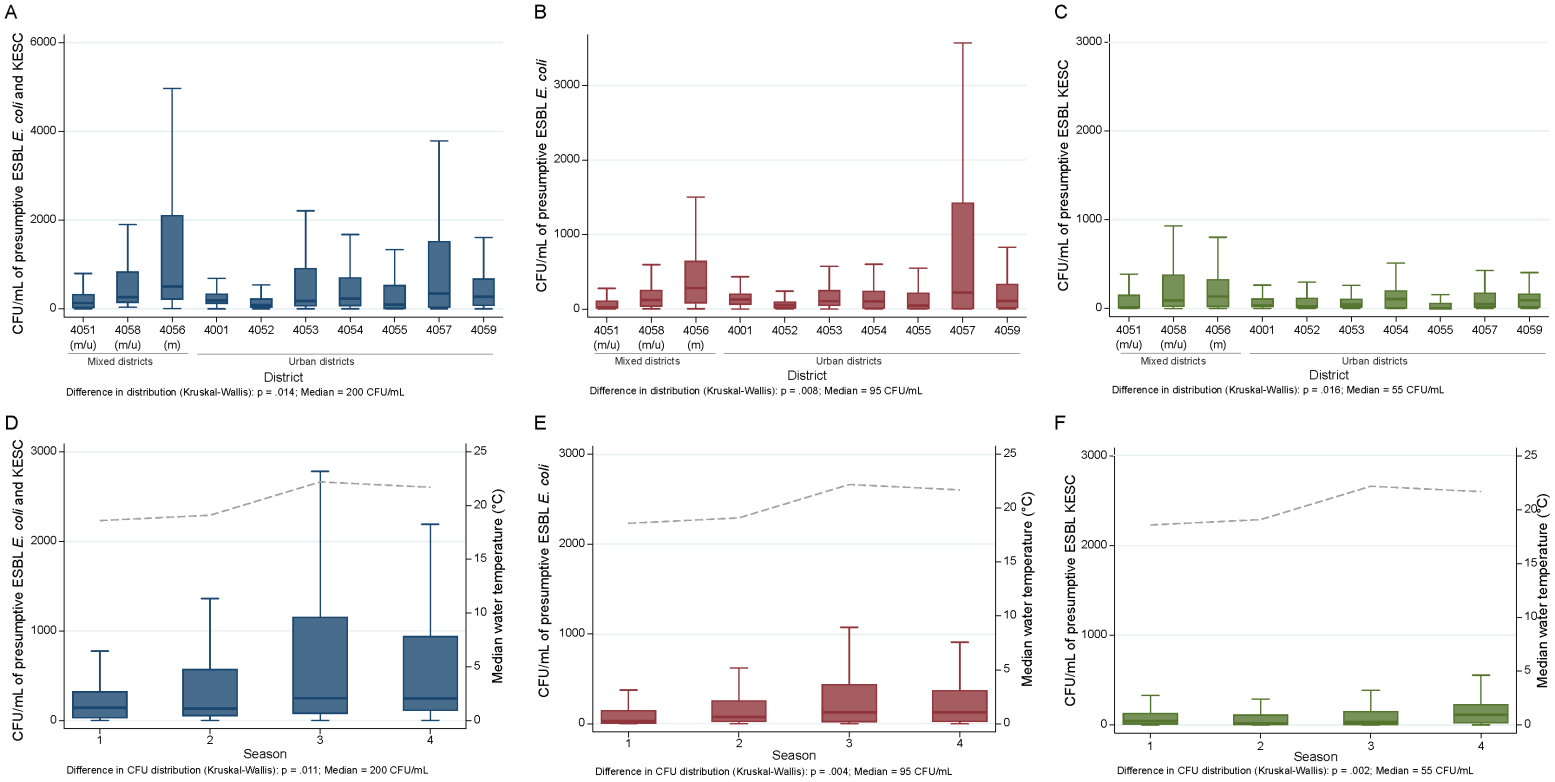
Spatiotemporal quantification of presumptive ESBL-producing *E. coli* and KESC groups. **(A–C)** Spatial distribution of presumptive ESBL-producing *E. coli* **(A)**, presumptive ESBL-producing KESC **(B)** and presumptive ESBL-producing *E. coli* plus KESC **(C)** across city districts and median population size. **(D–F)** Temporal distribution of presumptive ESBL-producing *E. coli* **(D)**, presumptive ESBL-producing KESC **(E)** and presumptive ESBL-producing *E. coli* plus KESC **(F)** across sampling seasons and median water temperature (stippled line). (m) mixed site, (u) urban site. 1, Winter; 2, Spring; 3, Summer; 4, Autumn.

Quantification of bacterial colony counts without pre-enrichment step was performed on 294 samples collected across 14 consecutive months (August 2018 to September 2019). Of these, 253, 227, and 273 samples harbored presumptive ESBL-producing *E. coli* (86.1%), KESC (77.2%) or at least one both groups (92.9%), respectively. Supplementary Table S6 displays all individual concentrations, overall statistics per sample across space and time as well as across urban/mixed sites for both species groups. Overall, a median of 2 × 10^2^ colony forming units per milliliter (CFU/mL) was detected. Presumptive ESBL-producing *E. coli* and KESC median counts were 9.5 × 10^1^ and 5.5 × 10^1^ CFU/mL, respectively, and differed across sites and sampling months (Supplementary Figure 1). Notably, district 4,056, which collected water from three referral hospitals, enclosed the overall highest CFU/mL numbers of resistant *E. coli* plus KESC (median 505 CFU/mL vs. 200 CFU/mL overall) species group (Figure 6 and Supplementary Table S6). The number of presumptive ESBL-producing *E. coli* and KESC was highest in August, when the median water temperature registered at sampling was also the highest (Supplementary Figures S1D–F). When analyzed by season, the numbers of CFUs were highest in summer for presumptive ESBL-producing *E. coli* and in autumn for KESC and differed across seasons (Figure 6 and Supplementary Table S6).

**Figure 7:**
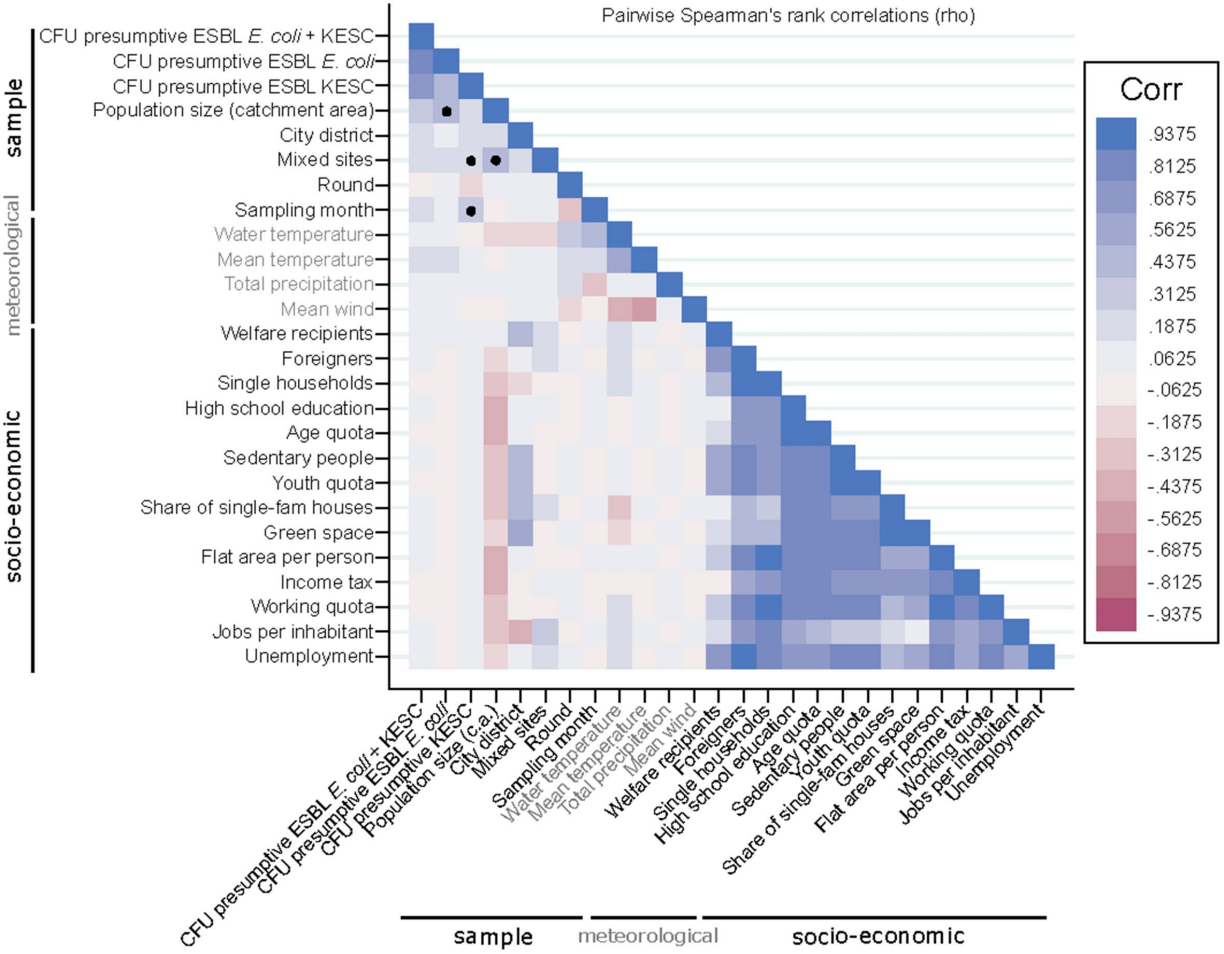
Correlation matrix of sample, meteorological and socio-economic parameters. Black dots denote the presumptive ESBL-producing *E. coli* and/or KESC-dependent positive correlations further analyzed and discussed.

**Figure 8:**
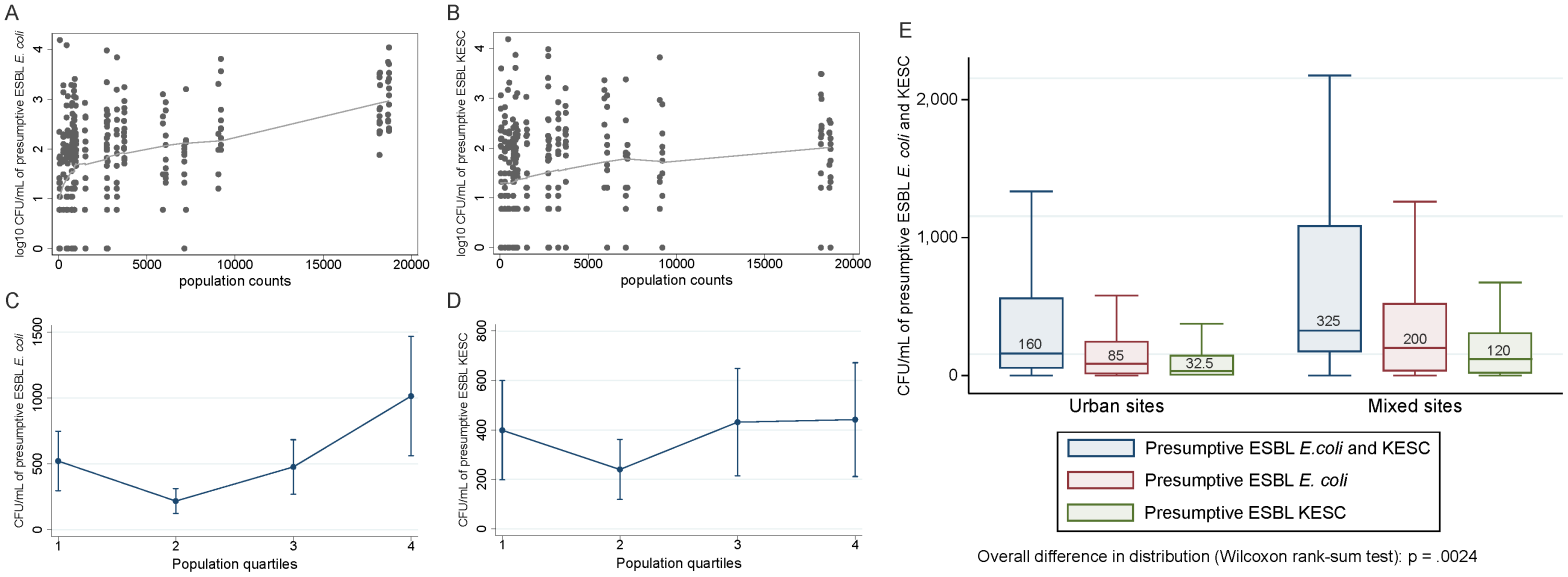
Analysis of relevant positive correlations involving presumptive ESBL-producing *E. coli* and/or KESC abundance. **(A,B)** Quantification of presumptive ESBL-producing *E. coli* **(A)** and presumptive ESBL-producing KESC **(B)** colonies across population counts. Trend lines with «Locally Weighted Scatterplot Smoothing» (LOWESS). **(C,D)** Predicted quantification based on negative binomial regression model (unadjusted) of presumptive ESBL-producing *E. coli* **(C)** and presumptive ESBL-producing KESC **(D)** with 95% confidence intervals. Minimum and maximum population size per quartile are as follows (quartile): (1) 39–724, (2) 744–972, (3) 993–3,727, (4) 5911–18,727. **(E)** Quantification of presumptive ESBL-producing *E. coli*, KESC and both groups in urban and mixed sites. Medians are indicated in each boxplot.

Correlation analyses were conducted to assess relationships between the quantification of both species groups and different socio-economic and meteorological variables (Figure 7 and Supplementary Table S7). We detected moderate correlations between population size and the number of presumptive ESBL-producing *E. coli*-CFUs (rho = 0.44), while KESC abundance was less related to population size (rho = 0.21) (Figures 8A–D). The median overall counts for mixed sites doubled those from the compiled urban sites (3.3 × 10^2^ vs. 1.6 × 10^2^ CFU/mL) (*p* = 0.002, Figure 8E) with greater differences in KESC counts (1.2 × 10^2^ vs. 3.3 × 10^1^ CFU/mL) as compared to *E. coli* counts (2.0 × 10^2^ vs. 8.5 × 10^1^ CFU/mL).

## Discussion

ESBL-producing *E. coli* and *K. pneumoniae* were detected in the vast majority of municipal wastewater samples of the city of Basel, Switzerland, over a two-year study period, indicating widespread dissemination of ESBL-PE in the community and supporting that community sources are important reservoirs entertaining the spread of ESBL-PE. Hospital-influenced abundance of ESBL-PE appears to be species dependent reflected by higher proportions and counts of *K. pneumoniae* but not *E. coli* in wastewater samples from sites receiving both hospital and community wastewater. This finding is further supported by the detection of a moderate correlation between ESBL-*E. coli*-counts in wastewater and population size, yet a weak correlation between counts of other ESBL-PE species and population size.

Our results deviate from the results of a study performed in France on wastewater samples collected in 2011, revealing significantly higher proportions of ESBL-production among *E. coli* isolates recovered from hospital as compared to general urban wastewater (7.5% vs. 0.1%) (Brechet et al., 2014). In line, higher proportions of ESBL-producers were detected among *E. coli* strains isolated from hospital effluents (37%) as compared to municipal sewage (18%) in Poland in a study published in 2013 (Korzeniewska et al., 2013). In addition, this study reported lower proportions of hospital effluents and inflow sewage samples being positive for ESBL-producing *E. coli* as compared to our study (76.5 and 57.1%). Both the French and the Polish studies point to hospitals being a more important reservoir for ESBL-*E. coli*, as compared to the community, over a decade ago. In line with our results and supporting the rising contribution from the community, a recent study on ESBL-producing *E. coli* in urban community wastewater from socio-spatially different communities in Germany also recovered phenotypic ESBL-producing *E. coli* from every wastewater sample across a one-year monthly-based sampling, demonstrating that the general community is an important indirect discharger (Schmiege et al., 2021). Although at a much lower scale, Kutilova and colleagues reported positive ESBL-producing *E. coli* in all samples tested across municipal and hospital wastewaters including different clearing stages from Czech Republic in 2016 (Kutilova et al., 2021). Recent publications from numerous European countries, such as Croatia, Hungary, Germany, Czech Republic, The Netherlands, Romania, Slovakia, Norway, and Sweden, evidence that AMR Enterobacterales, including ESBL-producing isolates, are spread throughout the different wastewater systems, even in low-endemic regions (Fagerstrom et al., 2019; Paulshus et al., 2019; Lepesova et al., 2020; Surleac et al., 2020; Hooban et al., 2021; Kutilova et al., 2021; Schmiege et al., 2021; Mutuku et al., 2022; Puljko et al., 2022).

All ESBL-PE recovered in our study belonged to either the Enterobacteriaceae (*E. coli*, *Klebsiella* spp., *Enterobacter* spp., *Citrobacter* spp., and *R. ornithinolytica*) or Morganellaceae (*M. morganii* and *P. mirabilis*) families. ESBL-producing *E. coli* was by far the most common and persistent species, followed by *K. pneumoniae* mirroring the epidemiology of ESBL-PE in clinical samples (Gasser et al., 2019; Antimicrobial Resistance Collaborators, 2022). In line with our results, a recent systematic review showed that the most common producer of ESBL in wastewater is *E. coli* (51/57 studies), followed by *Klebsiella* spp. (29/57 studies) and *Enterobacter* spp. (18/57 studies) (Zaatout et al., 2021).

Median levels of presumptive ESBL-producing *E. coli* (9.5 × 10^1^ CFU/mL wastewater) found in Basel were in line with those recently found among presumptive cefotaxime-resistant *E. coli* in influent wastewater in Croatia (average 1.7 × 10^1^–7.8 × 10^2^ CFU/mL) (Puljko et al., 2022) and between 1 and 10^4^ fold lower (10^3^–10^6^) than in additional European reports on community and hospital effluents (Korzeniewska et al., 2013; Brechet et al., 2014; Kwak et al., 2015; Hocquet et al., 2016; Schmiege et al., 2021; Mutuku et al., 2022). Nevertheless, comparative studies are limited as most available works focused on total coliforms or total *E. coli* (Lepesova et al., 2020). The number of presumptive ESBL-producing *E. coli* and KESC was highest in summer and in autumn, respectively, and differed across seasons. Further, numbers of presumptive ESBL-PE were highest in August, when the median water temperature registered at sampling was also the highest. These observations are supported by previous studies pointing to correlations between increasing outdoor temperatures and antibiotic resistant bacteria (MacFadden et al., 2018; Bock et al., 2022). This study cannot address if the increase observed was related to a selective increase of ESBL-PE or to an overall increase in bacterial counts, as the later was not measured.

Remarkably, ESBL-producing *R. ornithinolytica* was exclusively prevalent in sampling site 4,001/1, representing the city center. *R. ornithinolytica* is widely found in aquatic environments, insects and fishes (Sekowska, 2017). Infections by *R. ornithinolytica* are uncommon in humans but are increasing (Appel et al., 2021).

For some species recovered, i.e., *E. coli*, *K. pneumoniae*, and *R. ornithinolytica*, the selective media Brilliance™ ESBL agar, a validated commercial selective agar used for the identification of presumptive ESBL-producing *E. coli* and KESC group isolates (Blane et al., 2016; Lee et al., 2021), was an excellent predictor for ESBL-producing isolates (>97%). However, for other species, particularly for *E. cloacae* and *M. morganii*, >96% were ESBL negative. Interpretation of inhibition halos in the phenotypic test for these species suggests expression of inducible (or derepressed) endogenous AmpC β-lactamases, characteristic of these species. Hence, we found that enterobacterial species enclosing 3rd generation cephalosporin resistance mechanisms other than ESBL are consistently recovered. All isolates were recovered in the presence of cefpodoxime, a third generation cephalosporin. Blane, Brodrick (44) assessed the sensitivity and selectivity (suppressed growth of no ESBL producers) of ESBL-PE of Brilliance™ ESBL agar and ChromID ESBL agar, another gold standard media for this purpose that we also tested prior to the study, with and without pre-enrichment to detect ESBL-PE in stool samples. Both media performed comparable sensitivity and selectivity for all positive samples recovered. Pre-enrichment with cefpodoxime significantly increased sensitivity (59–98% for Brilliance™ ESBL agar) but reduced selectivity (87–61% Brilliance™ ESBL agar) in both agars, due to increased growth of non-ESBL-PE isolates. These results evidence our pre-enrichment-based isolation method was appropriate in prioritizing sensitivity and species diversity, while increased the range of false positive isolates, which we could be efficiently discriminated in subsequent analyses. On the other hand, the lack of pre-enrichment step for the quantification experiments had the additional effect of maximizing selectivity of ESBL-PE isolates, as no further species discrimination was pursued. We can then expect a higher proportion of ESBL-producing *K. pneumoniae* colonies within the KESC group with respect to the rate of non-ESBL producing *Enterobacter* spp. and *Citrobacter* spp. isolates recovered from the pre-enrichment counterpart, supporting the interpretation of our correlation results using the quantification data.

Our results support that both community and healthcare settings equally contribute to the spread of ESBL-PE. However, it is important to note that the proportion of ESBL-producing *K. pneumoniae* was higher in the mixed sites receiving hospital outlet water from several relevant hospitals in the city, while the proportion of ESBL-producing *E. coli* was higher in urban sites. In fact, district 4,056, which collected water from three referral hospitals, enclosed the overall highest CFU/mL numbers of resistant *E. coli* plus KESC species group. On top, quantification of presumptive ESBL-producing isolates enabled us to proxy resistant *K. pneumoniae* more abundant in mixed sites while having a lower dependency on population size than *E. coli.* The solid positive correlation observed between resistant *E. coli* and population size points to a direct and critical input from the household outlet water. Our results suggest a flow of species from different sources all contributing to the epidemiology of circulating ESBL-PE and reflect the importance of reducing concentrations of environmental pollutants, such as antibiotics and disinfectants, to diminish downstream spread. These findings support tailoring infection prevention and control measures in healthcare settings to the species of ESBL-PE as previously suggested (Tschudin-Sutter et al., 2012, 2017; Tacconelli et al., 2014) as the impact on the further spread of ESBL-producing *E. coli* may be less substantial as compared to the impact on other ESBL-PE-species.

A recent systematic review and meta-analysis on the prevalence of ESBL-PE in wastewater concludes that the prevalence of ESBL-PE in wastewater is increasing over time and that hospital wastewater is the most important repository of ESBL genes (Zaatout et al., 2021). Although antimicrobial resistance *E. coli* and ESBL-*E. coli* are commonly more abundant in untreated hospital wastewater than in community outlets (Hassoun-Kheir et al., 2020), there is consistent scientific evidence that the community represents a relevant source of ESBL-producing *E. coli*, making the effect of hospital effluent on ESBL-producing *E. coli* vague (generally, hospital effluents contribute less than 1% of overall municipal sewage) (Harris et al., 2013; Karkman et al., 2018; Kutilova et al., 2021; Schmiege et al., 2021). As shown in several European studies from Croatia, France, Poland, and Ireland (Galvin et al., 2010; Korzeniewska et al., 2013; Brechet et al., 2014; Puljko et al., 2022), current wastewater treatment processes reduce but do not eliminate all ESBL-PE isolates, and even the proportion of resistant to susceptible isolates released may increase. Thus, identifying sources contributing to the ESBL-PE epidemic is critical to develop targeted interventions.

Our study has some important limitations. First, results may only be generalizable to countries with a similar prevalence of ESBL-PE. In Switzerland approximately 10% of all clinical *E. coli* strains and *K. pneumoniae* strains are reported as being resistant to third generation cephalosporins, suggesting the presence of ESBLs.^4^ Second, our wastewater sampling scheme did not cover the entire population of the city, but only approximately half. However, the sites were chosen to be representative of the entire city, thus selection for areas with different socio-economic characteristics of the population is unlikely. Third, quantification of presumptive ESBL-PE was not carried out during the whole study period and only covered 12 months. Yet, our work covers one of the largest study periods and applies one of the more consistent sampling efforts as compared to former wastewater-based studies (Zaatout et al., 2021). Fourth, our exploratory comparisons and associated hypothesis tests should not be considered as confirmatory. Fifth, we do not present results of ESBL-genotypes. Ongoing investigations on the *resistome*, *mobilome* and *virulome* profile of all recovered isolates together with phylogenomic analyses will further set light on the circulating clones in the sewage pipeline of this European city. Preliminary analyses rule out the presence of carbapenemases in all isolates recovered from wastewater. Likewise, whole-genome comparisons with hospital and foodstuff ESBL-producing isolates recovered across the same study period (2017/2019) will undoubtedly contribute to improve our current understanding on the prevalence and clonal distribution of ESBL-PE in urban settings from a holistic One-Health perspective.

In conclusion, our findings indicate widespread dissemination of ESBL-PE, especially *E. coli*, within the population of a middle European city and support that community reservoirs are similarly important as healthcare-associated reservoirs in entertaining the spread of ESBL-PE. The hospital input of ESBL-producing *K. pneumoniae* seems to contribute to the higher proportions and load recovered from municipal sites collecting hospital wastewater. These results may help develop and implement efficient targeted interventions in the community and hospitals to reduce environmental spread of ESBL-producing clinically relevant species and circulating epidemic clones.

## Data availability statement

The original contributions presented in the study are included in the article/Supplementary material, further inquiries can be directed to the corresponding author.

## Author contributions

EG-S analyzed, interpreted the data, and wrote the first draft of the manuscript. CB conceived the study, collected the data, and critically revised the manuscript. JR generated the figures, performed the statistical analyses, and critically revised the manuscript. MA, LM, and RS contributed to data collection. RF and LE collected data and contributed to analysis of data. IS was responsible for the ESBL confirmatory testing of most isolates. AG-M, AE, and PH revised the manuscript. TS provided valuable input on the conceptualization of the study and revised the manuscript. LA-B contributed to data collection and interpretation of results and revised the manuscript. ST-S conceived and supervised the study, analyzed the data, and revised the manuscript. All authors reviewed and approved the final manuscript.

## Funding

This work was supported by the University Hospital Basel, the University of Basel, and the Swiss National Science Foundation (National Research Programme “Antimicrobial Resistance,” NRP 72, grant no. 407240_167060).

## Conflict of interest

The authors declare that the research was conducted in the absence of any commercial or financial relationships that could be construed as a potential conflict of interest.

## Publisher’s note

All claims expressed in this article are solely those of the authors and do not necessarily represent those of their affiliated organizations, or those of the publisher, the editors and the reviewers. Any product that may be evaluated in this article, or claim that may be made by its manufacturer, is not guaranteed or endorsed by the publisher.

## Footnotes

^1^ https://www.who.int/teams/environment-climate-change-and-health/water-sanitation-and-health/sanitation-safety

^2^ statistik.bs.ch

^3^ https://docs.meteoblue.com/en/meteo/data-sources/datasets#nems

^4^ www.anresis.ch

## Supplementary material

SUPPLEMENTARY FIGURE S1 Spatiotemporal quantification of presumptive ESBL-producing *E. coli* and KESC group colonies. **(A-C)** Spatial distribution across sampling sites among both groups **(A)**, presumptive ESBL-producing *E. coli* **(B)**, and presumptive ESBL-producing KESC **(C)**. **(D-F)** Temporal distribution across sampling month and median water temperature among both groups **(D)**, presumptive ESBL-producing *E. coli* **(E)**, and presumptive ESBL-producing KESC **(F)**. Sampling sites are ordered according to mixed and urban site distribution. Outliers were removed for readability. Boxes, bold lines and whiskers indicate the interquartile ranges, medians, and 1.5 times the interquartile range, respectively.
