# Supplementary figures and images for: Spatiotemporal dissemination of ESBL-producing Enterobacterales in municipal sewer systems: a prospective, longitudinal study in the city of Basel, Switzerland
Source: Front Microbiol. 2023 May 12;14:1174336. doi: 10.3389/fmicb.2023.1174336 (PMC10213686; doi:10.3389/fmicb.2023.1174336)

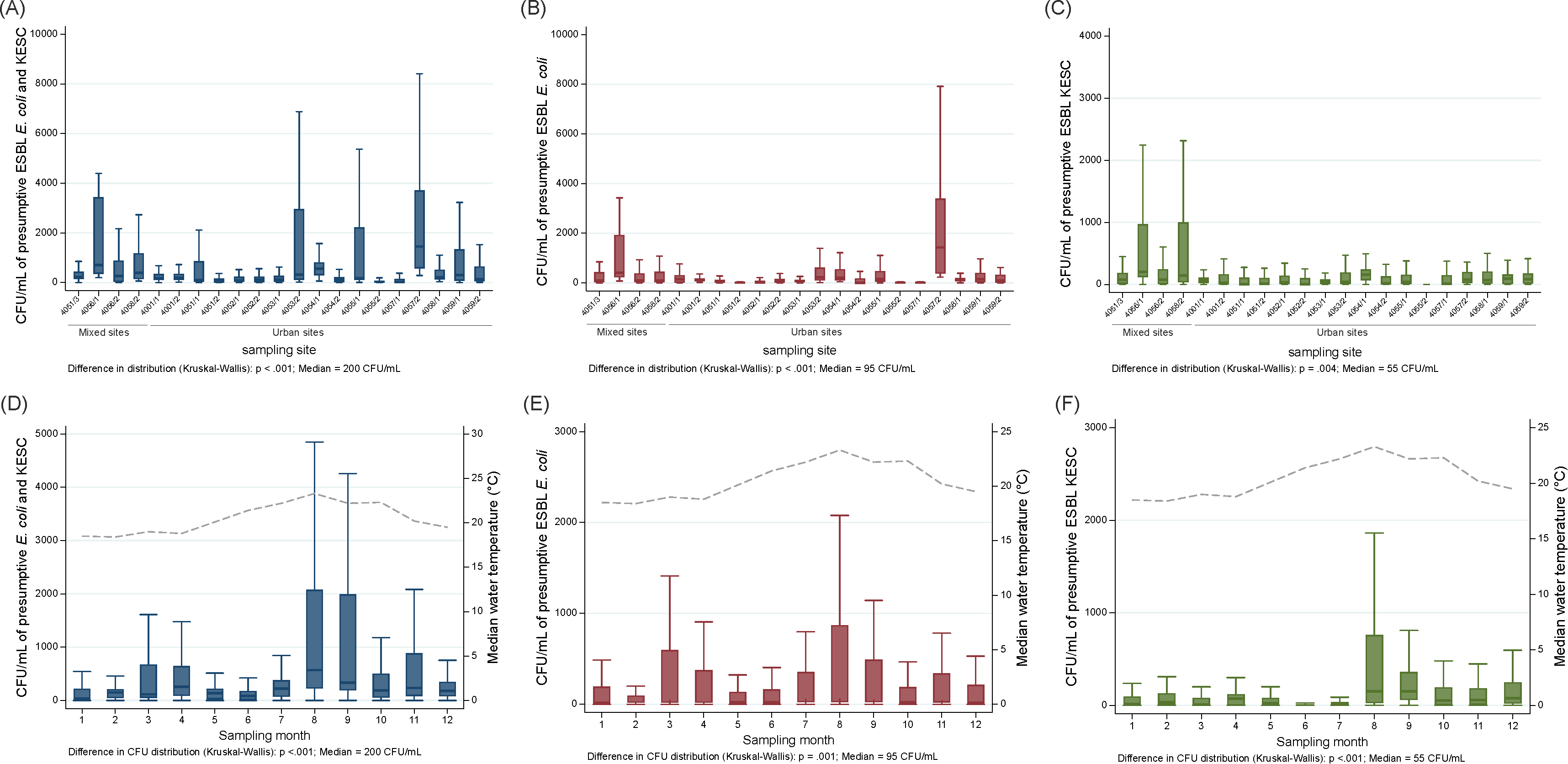

Supplement: SUPPLEMENTARY FIGURE S1 — Spatiotemporal quantification of presumptive ESBL-producing E. coli and KESC group colonies. (A-C) Spatial distribution across sampling sites among both groups (A), presumptive ESBL-producing E. coli (B), and presumptive ESBL-producing KESC (C). (D-F) Temporal distribution across sampling month and median water temperature among both groups (D), presumptive ESBL-producing E. coli (E), and presumptive ESBL-producing KESC (F). Sampling sites are ordered according to mixed and urban site distribution. Outliers were removed for readability. Boxes, bold lines and whiskers indicate the interquartile ranges, medians, and 1.5 times the interquartile range, respectively. [file Image_1.tif]
